# Study on parameter optimization of deep hole cumulative blasting in low permeability coal seams

**DOI:** 10.1038/s41598-022-09219-4

**Published:** 2022-03-24

**Authors:** Dan Zhao, Zhiyuan Shen, Minghao Li, Baichen Liu, Yinuo Chen, Lina Xie

**Affiliations:** 1 College of Safety Science & Engineering, Liaoning Technical University, Fuxin, 123000 China; 2 Key Laboratory of Mine Thermo-Motive Disaster and Prevention, Ministry of Education, Liaoning Technical University, Huludao, 125105 China; 3 College of Mechanical & Engineering, Liaoning Technical University, Fuxin, 123000 China; 4 Shenyang Institute of Technology, Shenyang, 110000 China

**Keywords:** Applied mathematics, Scientific data, Software, Engineering

## Abstract

Coal seam gas extraction is an important means of exploiting and utilizing gas resources, as well as a means of preventing coal mine disasters. To improve gas extraction efficiency in high gas and low permeability coal seams while ensuring blasting security, deep hole cumulative blasting parameters were optimized. ANSYS/LS-DYNA software is used to establish a 3-dimensional cumulative blasting model. By comparing and analyzing the blasting stress nephograms, stress time-history curves, and crack expansion curves, the optimal blasthole diameter, charge position, and charge length are obtained. Based on the numerical simulation results, a field test was carried out in the No. 10 coal seam of the Pingdingshan coal mine. The test results show that after cumulative blasting, the gas concentration was increased by an average of 2.25 times, the gas purity was increased by an average of 3.78 times, the permeability coefficient of the coal seam was increased by 21 times, and the effective radius of blasting was up to 7 m. The positive effects of deep hole cumulative blasting parameter optimization on the pressure relief and permeability enhancement of a high gas and low permeability coal seam were determined, which can provide a reference for other similar working faces to implement this technology.

## Introduction

Gas disaster is one of the main mine disasters affecting the safe of coal mines^1–4^. The most common method to prevent gas disaster is gas drainage^5–7^. Permeability enhancement is currently the most effective and economical gas drainage technology. Scholars have proposed measures such as presplitting blasting^8^, hydraulic fracturing^9^, liquid CO_2_ fracturing^10^, and loose blasting^11,12^ for improving permeability enhancement. Loose blasting is most widely used among them. Conventional blasting technology often encounters the difficult problems of poor coal seam fracture development or serious coal pulverization^13,14^. To improve the utilization rate of explosives, cumulative blasting technology has been proposed^15^. Cumulative blasting technology has the advantages of energy concentration and strong direction, which provides a new way to solve the problem of gas drainage.

In recent years, many scholars have carried out numerous research studies on cumulative blasting. In terms of studies on the fracturing mechanism, Guo et al.^16,17^ used ANSYS/LS-DYNA software to simulate the fracture mechanism and coal seam crack propagation from cumulative blasting, and the results indicate that the area around the blasting hole can be divided into the blast crush zone, blast fracture zone, and elastic deformation zone. Baêtaneves and Ferreira^18^ used the smooth particle hydrodynamics (SPH) algorithm to simulate the relationship between the angles of different cumulative hoods and the impact velocity of the cumulative jet. Zhao^19^ studied the mechanism of deep hole cumulative blasting and the laws of coal seam crack propagation by theoretical analysis, engineering experiments and numerical simulation experiments. In terms of studies involving engineering experiments, Lei et al.^20^ confirmed that the cumulative blasting is more effective in improving coal seam permeability through the comparative experiment of hydraulic fracturing and cumulative blasting. Liu et al.^21^ carried out laboratory experiments on cumulative blasting, which further confirmed the superiority of cumulative blasting to improve the permeability in Panyi coal mine. In terms of parameter optimization studies, the current researches are mainly aimed at drilling parameter optimization of cumulative blasting. Guo et al.^22,23^ studied the influence of blasting hole spacing, the distance between blasting hole and extraction hole, and the distance from blasting hole to roof and floor of the coal seam on blasting effect by cumulative blasting experiment in the Jiulishan coal mine. Song et al.^24^ proposed that when the decoupling coefficient of cumulative blasting radial charge is from 1.67 to 2, the permeability of coal seam is significantly improved.

Most studies on the fracturing mechanism and drilling parameter optimization of cumulative blasting regard cumulative blasting as a plane strain problem. However, in practice, different blasting parameters, such as charge length, will change the propagation characteristics of the detonation wave along the axis of the blasting hole, thereby affecting the blasting effect. Therefore, it is necessary to optimize the blasting parameters. In this paper, ANSYS/LS-DYNA software is used to establish 3-dimensional cumulative blasting models. From the angle of the plane perpendicular to the axis of the blasting hole, the influence of different blasting parameters on coal seam fracture development is analyzed. The optimal blasting hole diameter, charge position, and charge length are determined. According to the simulation results, the field test was carried out in the Pingdingshan No. 10 coal mine in China. The field test results were investigated. This paper has great significance for further improving coal seam permeability and provides experience for the application of cumulative blasting technology in coal mines.

## Theoretical basis of numerical simulation

### Principle of cumulative blasting

**Figure 1:**
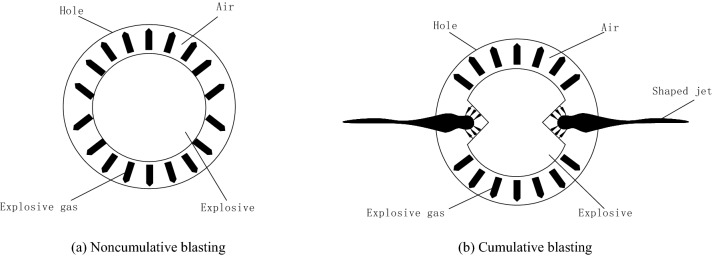
Principle of blasting action.

As shown in Fig. 1a, the pressure of the explosive gas produced by noncumulative blasting is evenly distributed, and the explosive gas spreads out in a nondirectional manner. This phenomenon leads to the small range of cracks produced by the explosion, and the effect is not obvious. As shown in Fig. 1b, the gas pressure generated by cumulative blasting is more concentrated in the cumulative direction, and the explosive gas concentrates on moving to the shaped charge tank. Most of the energy generated by the explosion is in the form of kinetic energy, so cumulative blasting better avoids energy dispersion and has a more obvious cracking effect.

### Numerical calculation method and boundary condition

The numerical simulation of cumulative blasting is a fluid–solid coupling process of the interaction between shaped charge flow, coal and air. Therefore, the ALE algorithm is used in the analysis, which can effectively combine the advantages of the Lagrange method and the Euler method to solve the large deformation calculation problem.

According to the needs of numerical simulation analysis, combined with the actual situation of the cumulative blasting field test, the following boundary conditions are imposed on the numerical model.

1. Upper boundary condition: Because the cumulative blasting test site is at a certain depth underground, the model must bear the weight of the overlying rock, so the upper boundary of the model is subjected to the gravity stress of the original rock. It can be expressed as follows^8,22^:
  1 $$q = \gamma gH$$
  where *q* is the top pressure (kN (m^2^)^−1^), *γ* is the average bulk density of the coal seam (N (m^3^)^−1^), *γ* is taken as 25 (m^3^)^−1^, *H* is the buried depth of the coal seam (m), and *H* is taken as 480 m.
  $$q = 480 \times 9.8 \times 25 = 117{,}600\,{\text{kN}}({\text{m}}^{2} )^{ - 1} = 117.6\,{\text{MPa}}$$
  To optimize the simulation, the pressure *q* on the upper boundary is set as 117.6 MPa.
2. Non reflection boundary condition: Because the test site was an infinite space and the size of the numerical simulation model was limited, the problem analysis could only be carried out in a limited area. Therefore, adding non reflection boundary conditions around the model could effectively eliminate the limitation of the model boundary.

### Constitutive model and material parameters

1. Constitutive mode and material parameters of the coal.
  In the calculation process, the elastic–plastic properties of coal and the changing strain rate should be considered, so the MAT_PLASTIC_KINEMATIC material model is used to simulate the constitutive relationship of coal under blasting conditions. The damage mode of the coal body under cumulative blasting is defined by the keyword MAT_ADD_EROSION. To meet the needs of numerical simulation, coal samples were taken in the Pingdingshan coal mine, and the physical and mechanical parameters of coal samples were measured through laboratory experiments. Detailed parameters are shown in Table 1.

**Table 1:** Physical and mechanical parameters of coal samples.

Name	Coal rock density	Elastic modulus	Poisson's ratio	Tensile strength	Compressive strength
Value	1.42	4.5	0.38	1.1	9.5
Unit	g/cm^−3^	GPa	–	MPa	MPa
2. Constitutive model and material parameters of the explosive.
  The material model of the explosive employed is MAT_HIGH_EXPLOSIVE_BURN, which corresponds to the JWL equation of state. The relationship between the pressure and the specific volume in the detonation process is given as^24^:
  2 $$P = A \left(1 - \frac{\omega }{{R_{1} V}}\right)e^{{ - R_{1} V}} + B \left(1 - \frac{\omega }{{R_{2} V}}\right)e^{{ - R_{2} V}} + \frac{{\omega E_{0} }}{V}$$
  where *P* is the detonation pressure (MPa), *A, B*,$\omega$, *R*_1,_ and *R*_2_ are explosive property constants, *V* is the mass volume of the gas products(m^3^), and *E*_0_ is the energy of the gas product when it explodes (MJ). The three-stage emulsion explosive is used in cumulative blasting in coal mines. Refer to reference^25^ for the calculation of explosive parameters, which will not be detailed in this paper. Explosive material parameters are shown in Table 2.

**Table 2:** Parameters of explosive material.

Name	Explosive density	Detonation velocity	Detonation pressure	*A*	*B*	*R*_1_	*R*_2_	$\omega$	E_0_
Value	0.9	2800	2.91	246.1	10.26	7.12	2.4	0.07	4.1
Unit	g/cm^3^	m/s	GPa	GPa	GPa	–	–	–	GPa

## Construction of the numerical model and results analysis

### Construction of the numerical model

**Figure 2:**
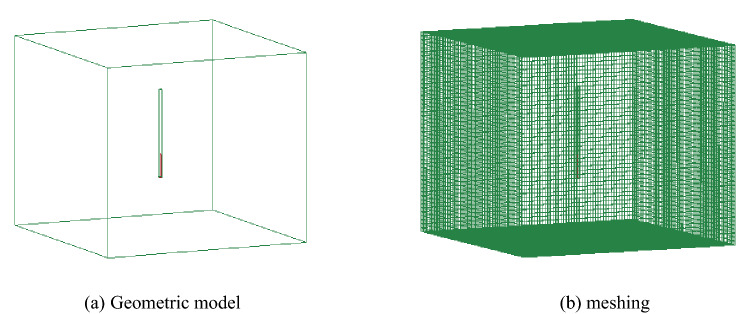
Model meshing.

The establishment of the numerical model was based on the actual situation of the F_15_-24100 working face in the No. 10 coal mine of Pingdingshan. The size of the numerical simulation model was 40 m × 40 m × 60 m. The hole depth and sealing length of the numerical model are 45 m and 12 m, respectively. The method of mapping and sweeping is used to divide the mesh. The number of units is 361,210, and the number of nodes is 378,970. As shown in Fig. 2, the charging position of this numerical model is at the bottom of the blasting hole, and the cumulative direction is the X plane direction.

### Simulation analysis of blasting hole diameter optimization

The radial uncoupled charge was used to simulate. According to the charge diameter of 42 mm and blasting holes of 89 mm and 94 mm used in the field test of the F_15_-24100 working face in the Pingdingshan coal mine, numerical simulation models of cumulative blasting with different diameters of 89 and 94 mm were established to optimize the blasting diameter. The blasting stress nephograms in the XY plane (cumulative direction) at T = 50 ms were extracted.

**Figure 3:**
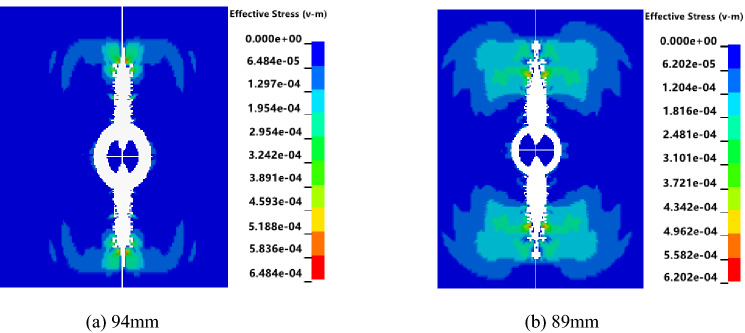
Blasting stress nephograms of different hole diameters.

Figure 3 shows that the stress of cumulative blasting on the coal body is different when the size of the blasting hole is different. In the cumulative direction, the stress range of the 89 mm aperture is larger than that of the 94 mm aperture. Two observation points, A and B, were selected in the effective influence area of the blasting hole. Point A was located in the cumulative direction. Point B was located in the noncumulative direction. A and B were 2 m away from the blasting hole. LS-PrePost was used for postprocessing analysis, and stress time-history curves of the two observation points were extracted.

**Figure 4:**
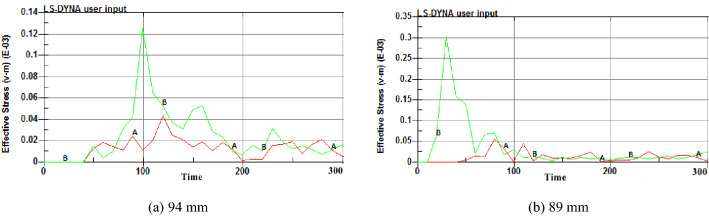
Stress time-history curves of different hole diameters.

Figure 4 shows that when the blasting diameter is 94 mm, the times for points A and B to reach the maximum stress are 124 ms and 99 ms, and the maximum stress values are 4.2 MPa and 12.3 MPa, respectively. When the blasting diameter is 89 mm, the times for points A and B to reach the maximum stress are 88 ms and 35 ms, and the maximum stress values are 5.1 MPa and 30.2 MPa, respectively. The stress value in the cumulative direction varies with the blasting diameter. When the blasting diameter is 89 mm, the stress peak in the cumulative direction is larger, and the time to reach the maximum stress is shorter. That is, an 89 mm blasting diameter makes coal more prone to fracture.

The maximum crack depths produced at different times and with different blasting diameters were extracted.

**Figure 5:**
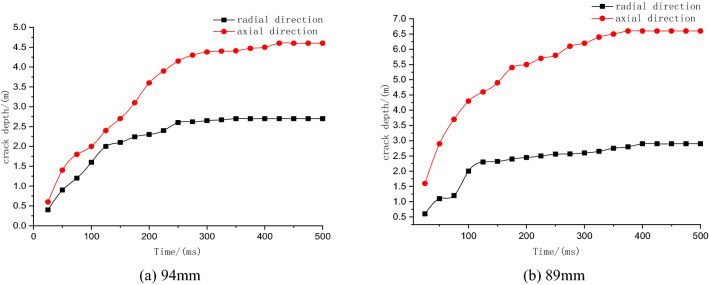
Crack expansion curves of different hole diameters.

Figure 5 shows that when the blasting diameter is 94 mm, the maximum crack depths in the axial (cumulative) and radial directions (noncumulative) are 4.6 m and 2.7 m, respectively. When the blasting diameter is 89 mm, the maximum crack depths in the axial and radial directions are 6.6 m and 2.9 m, respectively. In the cumulative direction, the cracks expand more completely, and the damage to the coal body is greater. In summary, a blasting diameter of 89 mm is the best choice.

### Simulation analysis of charging positions optimization

Numerical simulation models of cumulative blasting were established for different charging positions at the bottom, middle, and top of the blasting hole. The blasting stress nephograms in the YZ plane at T = 150 ms were extracted (the angle of plane perpendicular to the axis of the blasting hole).

**Figure 6:**
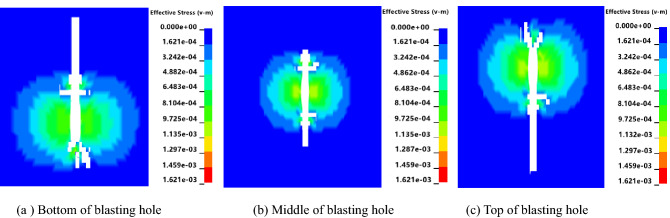
Blasting stress nephograms of different charging positions.

As shown in Fig. 6, when the charging position is at the bottom and top of the blasting hole, the upper and lower coal bodies are not subject to significant stress. When the charge position is in the middle of the blasting hole, the upper and lower coal bodies are under stress, and the range is larger. Five observation points, A, B, C, D, and E, were selected in the effective influence area of the blasting hole, and each point was 6 m apart. The horizontal distance from point C to the center of the explosive was 3 m. The stress time-history curves of the five observation points were extracted.

**Figure 7:**
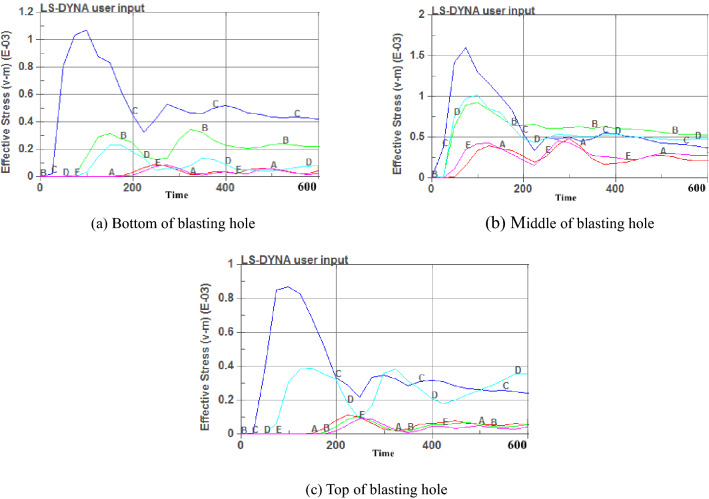
Stress time-history curves of different charging positions.

**Figure 8:**
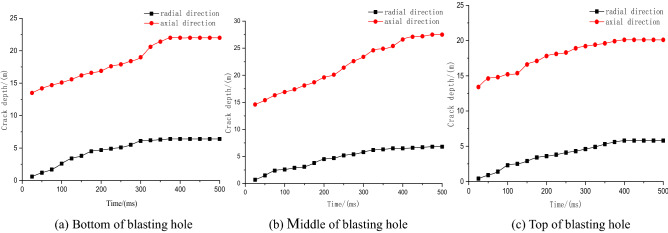
Crack expansion curves of different charging positions.

Figure 7 shows that different charging positions, and blasting of the effective region of the observation point time-history stress curve trends are generally the same. There are two stress peaks and the first stress peak is significantly larger than the second stress peak. This is because the explosives after the detonation of the cumulative jet first act on the coal body, resulting in greater stress, and then the blast wave and the air layer produce a second reflection wave on the coal body, resulting in a second stress peak. The peak stress at point C is different for different charging positions. The maximum stress value is approximately 154 MPa when the charge position is in the middle, and the time required to reach the maximum stress is the shortest, approximately 95 ms. When the charging position is in the middle, the stress values of the five observation points have changed greatly, while the stress values of points A and E change slightly when the charge position is at the top and bottom. When charging in the middle, the effective range of blasting is larger, and the stress action time is longer. Crack expansion curves of different charge positions are shown in Fig. 8.

Figure 8 shows that when the charging position is at the top and bottom of the blasting hole, the crack extension in the axial direction tends to stabilize at approximately 380 ms and 370 ms, and the maximum crack depths are 22 m and 77 m, respectively; the maximum crack depths in the radial direction are 6.4 m and 6.3 m, respectively. When the charging position is in the middle of the blasting hole, the crack extension in the axial direction tends to stabilize at approximately 450 ms, and the maximum crack depth is 27.5 m, which increases by 6.5 m on average compared with other forms. Therefore, a charging position in the middle of the blasthole is the best choice.

### Simulation analysis of charging length optimization

In blasting engineering, to improve the utilization rate of explosions and reduce the cost, an axial uncoupled charge is usually used. Therefore, choosing the appropriate charge length is essential to achieve a good blasting effect. According to the actual situation of the Pingdingshan coal mine, the charge length is usually 13 m and 20 m, so cumulative blasting models with charge lengths of 13 m and 20 m were established. The blasting stress nephograms in the YZ plane at T = 300 ms were extracted (the angle of the plane perpendicular to the axis of the blasting hole).

**Figure 9:**
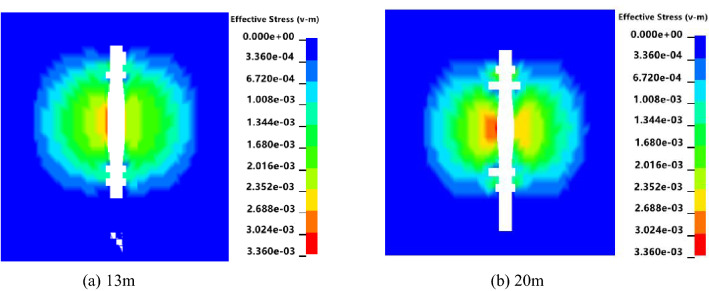
Blasting stress nephograms of different charging lengths.

Figure 9 shows that when the charging length is 20 m, the range of stress caused by the explosion is larger, and the center position stress of the blasting hole on the coal body is greater than 13 m. Five observation points, A, B, C, D, and E, were selected in the effective influence area of the blasting hole, and each point was 10 m apart. The horizontal distance from point C to the center of the explosive was 5 m. The stress time-history curves of the five observation points were extracted.

**Figure 10:**
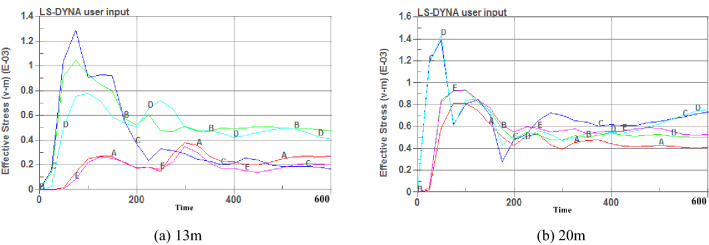
Stress time-history curves of different charging lengths.

**Figure 11:**
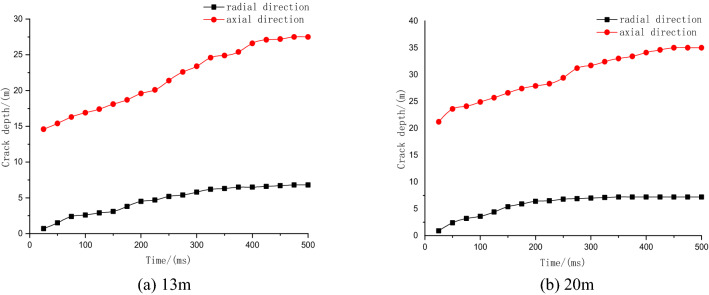
Crack expansion curves of different charging lengths.

Figure 10 shows that when the charge length is 13 m, point C reaches a peak stress of 128 MPa at 88 ms. The stress values of the five observation points eventually stabilized, with an average value of approximately 32 MPa. When the charge length is 20 m, point C reaches the peak stress of 146 MPa at 65 ms, the time is shortened by 23 ms, and the peak stress increases by 22 MPa. The stress value of the five observation points is stable at 60 MPa, which is approximately twice the length of the charge of 13 m. Crack expansion curves of different charge lengths are shown in Fig. 11.

Figure 11 shows that when the charge length is 13 m, the axial and radial crack expansion stabilizes at 450 ms, and the maximum axial and radial crack depths are 27.5 m and 6.8 m, respectively. When the charge length is 20 m, the maximum axial crack depth is 35 m, which is 1.3 times the charge length of 13 m, and the maximum radial depth is 7 m, which is 0.4 m more than the charge length of 13 m. Therefore, when the charge length is 20 m, the axial fissure depth is larger, the stress action range is wider, and the effective impact area of blasting is larger.

Based on the above numerical simulation results, it can be determined that the optimal parameters of cumulative blasting in the Pingdingshan coal mine are as follows: the blasting hole diameter is 89 mm, the charge length is 20 m, the charging position is in the middle of the blasting hole, and the effective blasting influence radius is 7 m.

## Field experiment of cumulative blasting

### Experimental conditions

Experiments were carried out at the F_15_-24100 working face of the No. 10 coal seam in the Pingdingshan coal mine. The thickness of the F_15_ coal seam is 2.5–3.5 m, and the average coal thickness is 3.2 m. The inclination angle of the coal seam is 10°to 12°, and the maximum original gas pressure value is 1.0 MPa, which shall be managed following the outburst coal seam. The gas content of the No.10 coal seam is 4.50–4.68 m^3^ t^−1^. It is measured on-site that the coal seam gas pressure of the F_15_-24100 working face is 2.51 MPa. The gas permeability coefficient is 0.076 m^2^ (MPa^2^ d)^−1^. The maximum raw coal gas content is 6.98 m^3^/t. The No.10 coal seam firmness coefficient is 0.48.

### Drilling layout of cumulative blasting experiment

**Figure 12:**
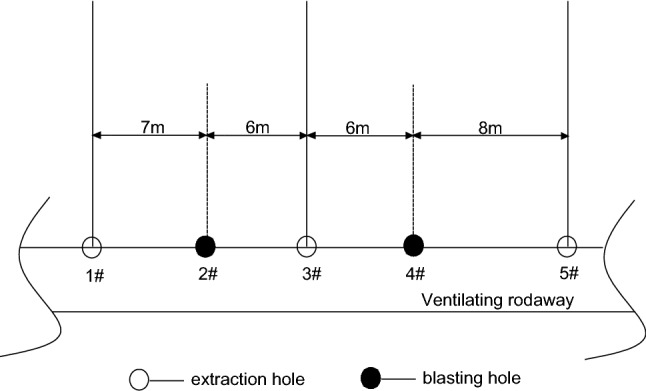
Schematic diagram of the drilling construction at the test site.

According to the numerical simulation results and the gas geological conditions of the F_15_-24100 working face in the Pingdingshan coal mine, parallel boreholes were drilled at a certain distance along the coal seam at the side of the air intake road. There were 5 boreholes in this test, among which #2 and #4 were blasting holes and the rest were extraction holes. The spacings between the blasting hole and the extraction hole were designed to be 6 m, 7 m, and 8 m. The layout of the drilling is shown in Fig. 12. From the numerical simulation results, it was determined that the hole diameter of the blasting hole was 89 mm, the charge length was 20 m, and the charge location was in the middle of the blasting hole. The larger the extraction hole diameter is, the more powerful the development of coal fractures. Therefore, an extraction hole diameter of 94 mm was selected. Drilling parameters are shown in Table 3.

**Table 3:** Drilling parameters.

Drilling number	Type	Aperture	Drilling depth	Charging length	Sealed hole length
#1	Extraction hole	94	80	–	–
#2	Blasting hole	89	45	20	12
#3	Extraction hole	94	80	–	–
#4	Blasting hole	89	45	20	12
#5	Extraction hole	94	80	–	–
Unit	–	mm	m	m	m

### Investigation of the experimental effect

**Figure 13:**
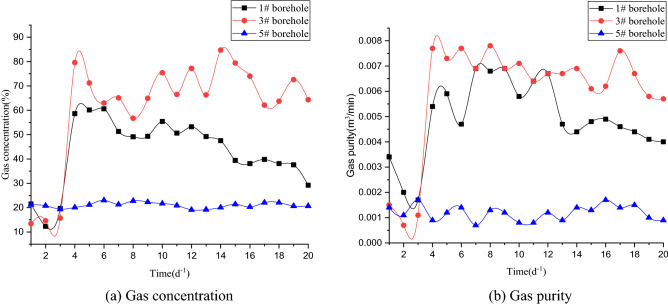
Gas parameter variation curves.

The most direct purpose of deep hole cumulative blasting is to produce a large number of cracks in the coal body to improve the permeability of the coal mass. Therefore, the increasing range of gas concentration and gas purity are important indicators to evaluate the effect of cumulative blasting in the Pingdingshan coal mine. After drilling, gas concentration sensors and gas flow sensors were installed in each extraction pipeline and used to monitor and record the gas concentration and gas purity in extraction holes from July 24 to August 12. The “yellow sand + yellow mud” joint sealing method was adopted. The cumulative blasting test was carried out on July 27, using the MFD-200 safety explosion-proof detonator. The observation time was 20 days. The variation curves of gas concentration and gas purity of each extraction hole are shown in Fig. 13. A comparison of the average gas concentration and gas purity before and after blasting is shown in Table 4.

**Table 4:** Comparison of monitoring data for extraction holes before and after blasting.

	Drilling number	Average gas concentration	Average gas purity
Before blasting	#1	28.8	0.0018
	#3	14.6	0.0011
	#5	20.5	0.0014
	Average	21.3	0.00143
After blasting	#1	65.0	0.0084
	#3	69.9	0.0069
	#5	21.1	0.0011
	Average	52.0	0.0054
	Unit	%	m^3^/min

It can be seen from Fig. 13 and Table 4 that before blasting, the gas concentration of extraction hole #1 fluctuated between 27.5 and 30.1%, with an average gas concentration of 28.8%. The gas purity varied between 0.0014 and 0.0023 m^3^/min, with an average gas purity of 0.0018 m^3^/min. After the implementation of deep hole cumulative blasting permeability increasing technology, the gas concentration and gas purity of extraction hole #1 were significantly improved. The gas concentration varied between 51.2 and 86.8%, with an average gas concentration of 65%. The gas purity varied between 0.0071 and 0.0097 m^3^/min, with an average gas purity of 0.0084 m^3^/min. Although the monitoring value decreased slightly in the following days, it was still higher than before blasting, which achieved an obvious effect of increasing the permeability. This shows that extraction hole #1 is within the effective influence range of blasting.

Before blasting, the gas concentration of extraction hole #3 fluctuated between 13.4 and 15.7%, with an average gas concentration of 14.6%. The gas purity varied between 0.0007 and 0.015 m^3^/min, with an average gas purity of 0.0011 m^3^/min. After cumulative blasting, the gas concentration and gas purity of extraction hole #3 were significantly improved. The gas concentration varied between 56.7 and 84.7%, with an average gas concentration of 69.9%. The gas purity varied between 0.0057 and 0.0078 m^3^/min, with an average gas purity of 0.0069 m^3^/min. Compared with extraction hole #1, the gas concentration and purity of extraction hole #3 have a larger variation. Considering that the hole has been blasted twice between blasting holes #2 and #4, the variation in gas concentration and purity in this hole changes greatly. It also indicates that increasing the number of blasting actions can significantly improve the effect.

The curve of gas concentration and purity in extraction hole #5 did not fluctuate significantly, and the average gas concentration and purity did not increase significantly. It can be explained that extraction hole #5 is not affected by blasting. The hole is not within the effective range of blasting. That is, the effective extraction radius of the cumulative blasting test is less than 8 m. Extraction hole #1 was in the effective range of influence being 7 m away from the blasting hole, while extraction hole #5 was not, it being 8 m away from the blasting hole. Therefore, it can be determined that the effective extraction radius of the blasting test is 7 m, which is consistent with the simulation results.

### Permeability coefficient change analysis

The coal seam gas pressure in extraction hole #3 was measured after sealing. After the pressure measurement was completed, the gas pressure was removed. The permeability coefficient of the coal seam after blasting is calculated after the gas flow of the borehole is stabilized. The calculation formula is shown in Table 5. ^10^ The calculation steps are as follows:

1. The gas content coefficient is calculated as follows:
  3 $$\alpha = X \cdot \gamma /P^{1/2}$$
  where *X* is the gas content (m^3^/t), *γ* is the bulk density of coal (t/m^3^), and *P* is the gas pressure (MPa).
  $$\alpha^{\prime} = 7.47 \times 1.2/0.24^{1/2} = 18.29{\text{m}}^{3} /{\text{m}}^{3} \cdot {\text{MPa}}^{1/2}$$
2. The exposed area of the blasting hole coal wall is calculated as follows:
  4 $$S = 2\pi \cdot r_{1} \cdot L$$
  where *r*_1_ is the radius of the blasting hole(m), and *L* is the length of the blasting hole (m).
  $$S^{\prime} = 2 \times 3.14 \times 0.047 \times 4.4 = 1.29 \; {\text{m}}^{2}$$
3. At time *t*, the blasting hole ratio flow rate is calculated as follows:
  5 $$q_{\Delta } = q_{t} /S$$
  where *q*_t_ is the natural gas flow rate of the borehole at self-draining time *t* (m^3^/min), and *S* is the exposed area of the blasting hole (m^3^).
  $$q_{\Delta }^{\prime } = 3.24/1.29 = 2.51\;{\text{m}}^{3} /({\text{m}}^{2} \; {\text{d}})$$
4. The coefficients A and B are calculated as follows:
  6 $$A = \frac{{q_{\Delta } r_{1} }}{{p_{0}^{2} - p_{1}^{2} }}, \quad B = \frac{{4 \times p_{0}^{1.5} }}{{\alpha \cdot r_{1}^{2} }}$$
  where *p*_0_ is the measured gas pressure(MPa), *p*_1_ is the gas pressure after pressure relief (MPa), *α* is gas content coefficient (${\text{m}}^{3}\; {\text{MPa}}^{{1/2}}$), *r*_1_ is the radius of the blasting hole(m), $q_{\Delta }$ is the blasting hole ratio flow rate.
  $$A^{\prime} = \frac{2.51 \times 0.047}{{0.42^{2} - 0.1^{2} }} = 0.71 \quad B^{\prime} = \frac{{4 \times 0.42^{1.5} }}{{18.29 \times 0.047^{2} }} = 27.2$$
5. The permeability coefficient λ is calculated as follows:
  $$\lambda^{\prime} = 1.1A^{\prime}{1.25} \cdot B^{\prime}{0.25} = 1.63\;{\text{m}}^{2} \cdot ({\text{MP}}a^{2} \;\; {\text{d}})^{-1}$$
6. The time criterion F_0_ is verified as follows:
  $$F^{\prime} = B^{\prime} \cdot \lambda^{\prime} = 27.2 \times 1.63 = 44.34$$

**Table 5:** Formula table for the calculation of the permeability coefficient.

Flow rate Y	Time criterion	Permeability coefficient	Coefficient A	Coefficient B
$Y = aF_{0}^{b}$ $Y = \frac{A}{\lambda }$	10^–2^ to 1	$\lambda = {\text{A}}^{{{1}{\text{.61}}}} {\text{B}}^{0.61}$	$A = \frac{qr}{{p_{0}^{2} - p_{1}^{2} }}$	$B = \frac{{4 \times p_{o}^{1.5} }}{{\alpha r^{2} }}$
	1 to 10	$\lambda = {\text{A}}^{{{1}{\text{.39}}}} {\text{B}}^{0.39}$		
	10 to10^2^	$\lambda = 1.10{\text{A}}^{{{1}{\text{.25}}}} {\text{B}}^{0.25}$		
	10^2^ to 10^3^	$\lambda = 1.83{\text{A}}^{{{1}{\text{.14}}}} {\text{B}}^{0.14}$		
	10^3^ to 10^5^	$\lambda = 2.10{\text{A}}^{{{1}{\text{.11}}}} {\text{B}}^{{{0}{\text{.11}}}}$		
	10^5^ to 10^7^	$\lambda = 3.14{\text{A}}^{{{1}{\text{.07}}}} {\text{B}}^{0.07}$
  The verification result $F^{\prime}$ is between 10 and 10^2^, so the calculation formula for the air permeability coefficient is selected correctly. According to the calculation, after deep hole cumulative blasting, the permeability of the original coal seam is increased from 0.076 to 1.63 m^2^ (MPa^2^ d)^−1^, which is an increase of more than 21 times, and the permeability of the coal seam is greatly improved.

## Result and discussion

Before cumulative blasting experimental, the average gas concentration and purity of these extraction holes were 21.3% and 0.00143 m^3^/min, respectively. After the implementation of deep hole cumulative blasting at the F_15_-24100 working face of the NO. 10 coal seam in Pingdingshan, the average gas concentration and purity were 52.0% and 0.0054 m^3^/min, respectively. The average gas concentration increased by 2.25 times. The average gas purity increased by 3.78 times. The coal seam permeability increased from 0.076 to 1.63 m^2^ (MPa^2^ d)^−1^, increasing by more than 21 times.

The results show that new irreversible cracks are produced by cumulative blasting. The cracks in the coal body are extended, which greatly improves the amount of gas extraction and permeability coefficient in the coal seam. This shows that deep hole cumulative blasting technology is effective in promoting gas drainage in the Pingdingshan coal mine.

## Conclusion

1. ANSYS/LS-DYNA software is used to establish 3-dimensional cumulative blasting models. From the angle of the plane perpendicular to the axis of the blasting hole, the influence of different blasting parameters on the coal seam fracture development is analyzed. The simulation results of cumulative blasting show that a blasting hole diameter of 89 mm, a charge length of 20 m, and a charging position in the middle of the blasting hole can improve the effect of permeability enhancement technology. The blasting impact radius is determined to be approximately 7 m.
2. Field tests of cumulative blasting with different blasting parameters are carried out in the Pingdingshan coal mine. The average gas concentration increased from 21.3 to 52.0%, increasing by 2.25 times. The average gas purity increased from 0.00143 to 0.0054 m^3^/min, an increase of 3.78 times. The No.10 coal seam permeability increased from 0.076 to 1.63 m^2^ (MPa^2^ d)^−1^, increasing by more than 21 times. The effective extraction radius of the test is 7 m, which is consistent with the simulation results. The results of cumulative blasting parameter optimization have certain guiding significance.
3. This paper focuses on the optimization of blasting parameters. However, parameters such as the initial fracture, water content, and burial depth might affect the permeability enhancement. In a following study, we will focus on the influence of the above three parameters on the technology of cumulative blasting.
